# Knowledge Gaps in Rodent Pancreas Biology: Taking Human Pluripotent Stem Cell-Derived Pancreatic Beta Cells into Our Own Hands

**DOI:** 10.3389/fendo.2015.00194

**Published:** 2016-01-14

**Authors:** Munirah Mohamad Santosa, Blaise Su Jun Low, Nicole Min Qian Pek, Adrian Kee Keong Teo

**Affiliations:** ^1^Stem Cells and Diabetes Laboratory, Discovery Research Division, Institute of Molecular and Cell Biology, Singapore; ^2^School of Biological Sciences, Nanyang Technological University, Singapore; ^3^Department of Biochemistry, Yong Loo Lin School of Medicine, National University of Singapore, Singapore; ^4^Lee Kong Chian School of Medicine, Nanyang Technological University, Singapore

**Keywords:** pancreas, islet, beta cell, human, pluripotent stem cell

## Abstract

In the field of stem cell biology and diabetes, we and others seek to derive mature and functional human pancreatic β cells for disease modeling and cell replacement therapy. Traditionally, knowledge gathered from rodents is extended to human pancreas developmental biology research involving human pluripotent stem cells (hPSCs). While much has been learnt from rodent pancreas biology in the early steps toward Pdx1^+^ pancreatic progenitors, much less is known about the transition toward Ngn3^+^ pancreatic endocrine progenitors. Essentially, the later steps of pancreatic β cell development and maturation remain elusive to date. As a result, the most recent advances in the stem cell and diabetes field have relied upon combinatorial testing of numerous growth factors and chemical compounds in an arbitrary trial-and-error fashion to derive mature and functional human pancreatic β cells from hPSCs. Although this hit-or-miss approach appears to have made some headway in maturing human pancreatic β cells *in vitro*, its underlying biology is vaguely understood. Therefore, in this mini-review, we discuss some of these late-stage signaling pathways that are involved in human pancreatic β cell differentiation and highlight our current understanding of their relevance in rodent pancreas biology. Our efforts here unravel several novel signaling pathways that can be further studied to shed light on unexplored aspects of rodent pancreas biology. New investigations into these signaling pathways are expected to advance our knowledge in human pancreas developmental biology and to aid in the translation of stem cell biology in the context of diabetes treatments.

## Introduction

In the field of stem cells and diabetes, many scientists are actively pursuing the generation of insulin-secreting pancreatic β cells from human pluripotent stem cells (hPSCs) for β cell transplantation/replacement and treatment of diabetes (1–3). While insights from rodent pancreas developmental biology has guided the generation of PDX1^+^ pancreatic progenitors from hPSCs, the specific developmental principles thereafter remain murky. Henceforth, research groups have relied upon the transplantation of pancreatic progenitors, derived *in vitro*, into rodents for *in vivo* maturation (4–6). However, there has been considerable progress toward the generation of mature and functional human pancreatic β cells *in vitro* in the recent years. These β cells purportedly co-express cardinal β cell markers, such as PDX1, NKX6.1, musculoaponeurotic fibrosarcoma oncogene homolog A (MAFA), prohormone-processing enzymes, insulin, and C-peptide. Importantly, they are also monohormonal and glucose responsive.

Developmental biologists believe that there is much to be learnt from rodent developmental biology to guide hPSC-based generation of clinically useful cell types, such as pancreatic β cells. Owing to such efforts, the progression of definitive endoderm (DE) germ layer to PDX1^+^ pancreatic progenitors has been well-explored. However, the investigations on the later steps of pancreatic endocrine development and β cell maturation have not been quite fruitful. The most substantial advances in stem cell biology have relied upon an arbitrary approach of iterative trial-and-error testing to achieve mature and functional pancreatic β cells *in vitro* (7). Therefore, several pertinent questions remain: why were we not able to extrapolate rodent developmental principles and apply them on hPSCs to derive mature and functional pancreatic β cells? Are there differences between rodent and human pancreas development that prevent such an application? In this review, we look at signaling pathways that have been activated or repressed in stem cell biology and retrospectively revisit existing knowledge about rodent pancreas biology. Our efforts highlight novel aspects of signaling pathways that can be further investigated in our translational efforts for diabetes.

## Inhibition of Transforming Growth Factor-β Signaling in the Later Stages of Pancreatic Differentiation

**Figure 1:**
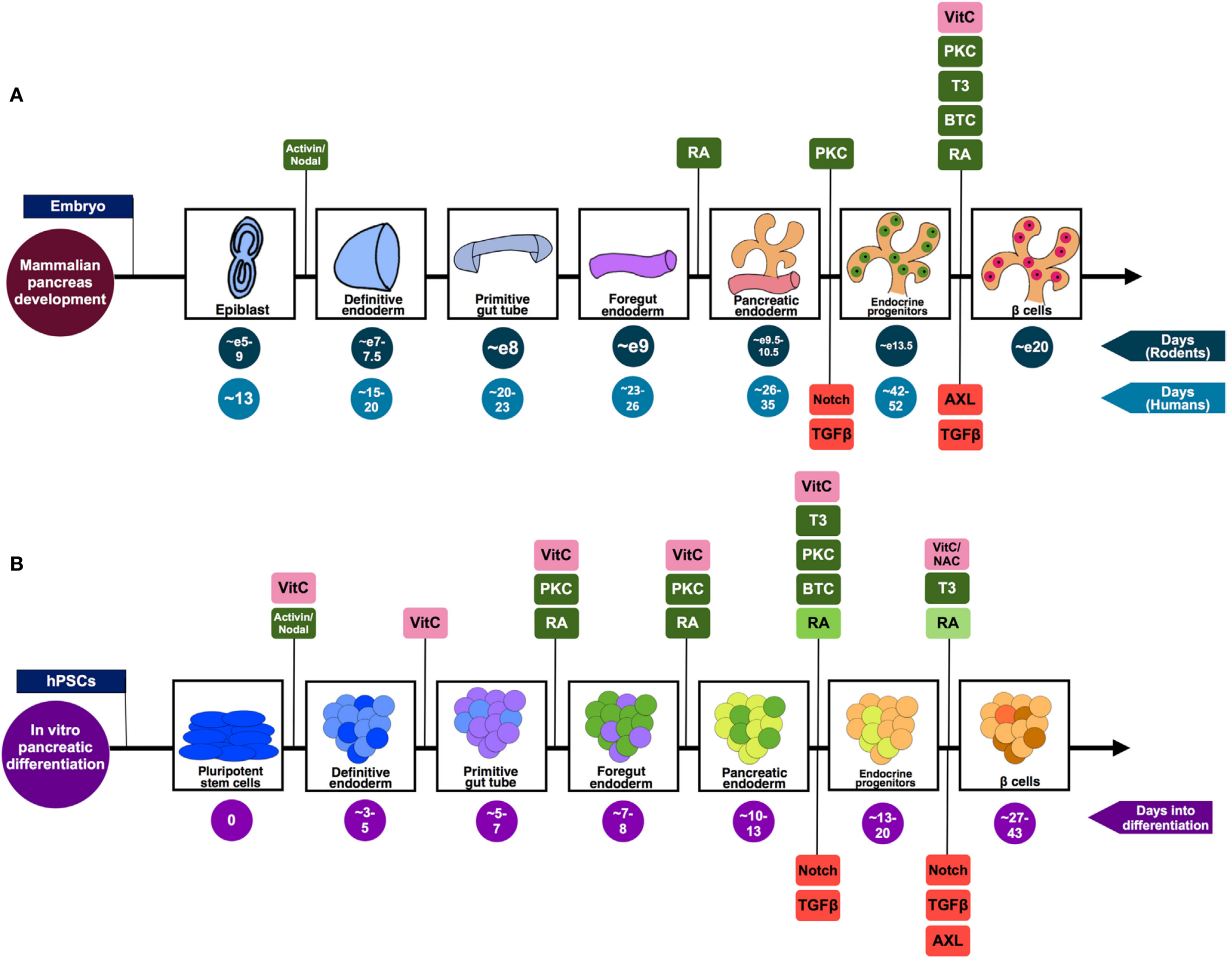
**A summary of the pivotal stages of pancreatic differentiation**. **(A)** Mammalian pancreas development based on knowledge from both rodent and human studies. The signaling pathways suggested to be positively regulating the differentiation process are highlighted in green, while those found to be inhibited in order to drive differentiation toward pancreatic lineage are shown in red. **(B)** An *in vitro* pancreatic differentiation timecourse generating β-like cells from hPSCs. The signaling pathways (green) and antioxidants (pink) that are positively regulating the differentiation process at each phase of development are highlighted. Stage-specific signaling pathways that are inhibited to drive differentiation toward pancreatic lineage are shown in red. The decreasing doses of RA used during the differentiation process [as described by Rezania et al. (6) and Pagliuca et al. (7)] are represented by the decreasing shades of green. The differing colors co-existing in an aggregate illustrates the heterogeneity of cells prevalent in such a differentiation scheme. While some of the cells will transit from being endocrine progenitors (light orange) to Pdx1^+^ insulin-producing β-like cells (brown), the end-product will include an assortment of maturing endocrine cell types (represented by orange and light orange).

The transforming growth factor-β (TGF-β) superfamily of proteins regulates pancreas development and function (8). TGF-β1, TGF-β2, and TGF-β3 are expressed in pancreatic epithelial cells at E12.5 in mice. Thereafter, they become localized in the acinar cells (9). TGF-β1 can promote the development of mouse pancreatic β cells from pancreatic buds (10). Perplexingly, it also indirectly inhibits the formation of mouse pancreatic epithelial cells (11). In tandem, TGF-β2 has been demonstrated to inhibit *Hnf1*β and *Pdx1* gene expression. Hence, TGF-β can purportedly restrain the specification of pancreatic cell fate (12). TGF-β signaling effector SMAD3 can bind the *Ins* gene promoter to suppress its expression. In agreement, *Smad3*-deficient islets exhibit an active insulin signaling pathway (13). Collectively, these evidences suggest the requirement to inhibit TGF-β signaling for the derivation of mature and functional pancreatic β cells (Figure 1A).

In 2011, Nostro et al. used small molecule SB431542 (14), an Activin/TGF-β receptor antagonist, in their pancreatic differentiation protocol. SB431542 inhibits activin receptor-like kinases (ALK) 4/5/7 and the downstream TGF-β/Activin/Nodal signaling. SB431542 treatment was demonstrated to increase *INS* gene expression and the development of C-peptide^+^ cells (15). Similarly, Cho et al. also utilized SB431542, in the presence of retinoic acid (RA), for pancreatic differentiation (16). Alternatively, Schulz et al. used TGF-βRI kinase inhibitor IV to obtain pancreatic progenitors from CyT49 hPSCs (17). Rezania et al. identified that the use of 2-(3-[6-Methylpyridin-2-yl]-1*H*-pyrazol-4-yl)-1,5-naphthyridine (ALK5iII) can effectively induce the expression of *NGN3*, *NEUROD1*, *INS*, and *GCG* transcripts to promote pancreatic endocrine specification (18). Rezania et al. further demonstrated that 1 μM ALK5iII is necessary for the induction of NEUROD1^+^ cells, but it suppressed the proportion of NKX6.1^+^ cells (4), a hallmark of functional β cells (19). Most recently, Rezania et al. compared the effects of several ALK5 inhibitors at a later phase of differentiation of hPSCs and found that only ALK5iII downregulated *NGN3* while increasing *INS*, *GCG*, and *SST* transcripts (6). Furthermore, 10 μM ALK5iII induced the expression of nuclear v-maf MAFA transcript, a critical mature β cell transcription factor, in diabetic rodents (20–22). Rezania et al. (6) concluded that ALK5iII was the most effective and specific inhibitor as it inhibited ALK5 but had minimal inhibition of other kinases. Similarly, Pagliuca et al. also employed 10 μM Alk5iII to derive mature and functional human pancreatic β cells from hPSCs (7) (Figure 1B; Table 1).

**Table 1:** **Summary of some novel signaling pathways perturbed during pancreatic differentiation of hPSCs**.

	Molecules	Mechanism	Induction of pancreatic lineage markers	Reference	Humans	Rodents
TGF-β inhibition	SB431542	Inhibits ALK 4/5/7	Upregulates *INS* gene expression and C-peptide^+^ cells	Nostro et al. (15)	√	
	SB431542 + RA		Upregulates *PDX1* gene expression	Cho et al. (16)	√	
	TGF-βRI kinase inhibitor IV		Induces pancreatic progenitors from hPSCs	Schultz et al. (17)	√	
	1 μM ALK5iII	Inhibits ALK5	Downregulates *NGN3*. Upregulates *NEUROD*, *INS*, *GCG*, and *SST* transcripts Induces *Mafa* transcript expression in diabetic rodents	Rezania et al. (4, 6, 18)	√	√	
	10 μM ALK5iII			Pagliuca et al. (7)	√

Protein kinase C signaling	300 nM ILV	Activates PKC Synergizes with FGF10 signaling	Upregulates gene expression of pancreatic progenitor markers *SOX9*, *PDX1*, *PTF1A*, *HNF6*, *PROX1* Upregulates gene expression of endocrine progenitor markers including *NGN3*, *NKX2.2*, and *NKX6.1*	Chen et al. (26)	√	√	
	500 nM TPB		Upregulates protein expression of FOXA2, PTF1A, HNF6, and NKX6.1		√	
	14 nM PMA		Downregulates protein expression of endoderm markers CDX2 and AFP		√	
	50 nM TPB	Activates PKC	Upregulates gene expression of pancreatic lineage markers *NGN3*, *NEUROD1*, *PTF1A*, and *NKX6.1* Downregulates gene expression of intestinal marker *CDX2* and liver marker *ALB*	Rezania et al. (4)	√	
	500 nM PDBu			Pagliuca et al. (7)	√	

Low retinoic acid (RA) signaling	1–3 μM RA	Activates RA receptors		Various	√	
	3 nM TTNPB			Schulz et al. (17)	√	
	1 μM → 100 nM → 50 nM RA			Rezania et al. (7)	√	
	2 μM → 100 nM → 25 nM RA			Pagliuca et al. (7)	√	

γ-secretase/Notch inhibitor	DAPT	Inhibits Notch signaling	Upregulates *NGN3* mRNA and protein expression in adult islets	Dror et al. (36)	√	√
			Upregulates *NGN3* and *NEUROD1* gene expression	Rezania et al. (18)	√	
	GSiXX		Upregulates expression of β cell maturation genes Downregulates expression of pancreatic exocrine marker *PTF1A*	Rezania et al. (6)	√	
	GSiXX + T3		Upregulates NKX6.1^+^insulin^+^GCG^-^ β-like cells	Rezania et al. (6)	√	
	1 μM XXI		Upregulates β cell gene expression	Pagliuca et al. (7)	√	

T3	0.1 μM T3	Activates MAPK/ERK signaling pathway	Induces rodent pancreatic β cell proliferation	Kim et al. (42)		√
	1 μM T3		Upregulates expression of *INS* and mature β cell markers	Rezania et al. (6)	√	
			Enhances co-expression of NKX6.1 and INS protein	Pagliuca et al. (7)		

AXL	2 μM BGB324 (R428)	Inhibits AXL	Upregulates MAFA protein expression	Rezania et al. (6)	√	
	GAS6	Activates AXL	Downregulates *Mafa* gene expression	Haase et al. (48)		√

Antioxidants	GPx-1	Antioxidants	Maintains protein expression of nuclear MAFA in diabetic rodents	Harmon et al. (22)		√
	NAC			Harmon et al. (21)		√
	Ebselen			Mahadevan et al. (20)		√
	1–2 mM NAC		Upregulates nuclear MAFA protein expression	Rezania et al. (6)	√	
	0.25 mM vitamin C		Generates mature and functional human pancreatic β cells	Pagliuca et al. (7)	√	

Betacellulin	BTC	Binds to ErbB-1 and ErbB-4 receptors to initiate PI3K/Akt, MAPK, STAT, and mTOR signaling pathways	Upregulates insulin secretion	Dahlhoff et al. (65)		√
			Upregulates mRNA and protein expression of IRS-2	Oh et al. (68)		√
			Induces *Pax4* gene expression in rat islets	Brun et al. (70)		√
			Sustains *PDX1* expression and induces β cell differentiation from hESCs	Cho et al. (71)	√
	10 ng/ml BTC		Upregulates *Pdx1* gene expression and insulin production Downregulates amylase and glucagon production in mouse embryonic pancreas explants	Thowfeequ et al. (72)		√
	20 ng/ml BTC		Induces pancreatic differentiation	Pagliuca et al. (7)	√
	50 ng/ml EGF		Preserves cell mass	Schulz et al. (17)	√

*BTC, Betacellulin; DAPT, *N*-[*N*-(3,5-difluorophenacetyl-l-alanyl)]-*S*-phenylglycine t-butyl ester; GPx-1, glutathione peroxidase-1; GSiXX, γ secretase inhibitor XX; ILV, (−)-indolactam V; NAC, *N*-acetylcysteine; PDBu, phorbol 12,13-dibutyrate; PMA, phorbol-12-myristate-13-acetate; T3, l-3,3′,5-Triiodothyronine; TPB, [(2S,5S)-(E,E)-8-(5-(4-(trifluoromethyl)phenyl)-2,4-pentadienoylamino) benzolactam]*.

Overall, the inhibition of ALK5/TGF-βRI with ALK5iII appears to be more desirable as compared to the general inhibition of TGF-β signaling via the use of SB431542. Further studies are certainly required to investigate the intricacies of TGF-β signaling during pancreas development and β cell maturation.

## Protein Kinase C Signaling Enhancement

Protein kinase C (PKC) is a family of serine/threonine kinases that are involved in diverse cellular processes, including survival, apoptosis, cell cycle regulation, proliferation, migration, and differentiation (23). In maturing neonatal rat islets, PKCα was only found in β cells, PKCγ in α cells, and PKCϵ in δ cells (24). This differential expression of PKC isoenzymes (25) hints that PKC signaling may play a role in the functional maturation of pancreatic endocrine progenitors (Figure 1A).

Chen et al. (26) was the first to demonstrate that 300 nM (−)-indolactam V (ILV) or PKC agonists {500 nM [(2*S*,5*S*)-(*E*,*E*)-8-(5-(4-(trifluoromethyl)phenyl)-2,4-pentadienoylamino) benzolactam (TPB)] or 14 nM phorbol-12-myristate-13-acetate (PMA)} can efficiently increase the formation of PDX1^+^ pancreatic progenitors from hPSCs via the activation of PKC signaling. ILV treatment resulted in an increased gene expression of several pancreatic progenitor markers, including *SOX9*, *PDX1*, *PTF1A*, *HNF6*, and *PROX1*, and endocrine progenitor markers, including *NGN3*, *NKX2.2*, and *NKX6.1*. The protein expression of pancreatic progenitor markers FOXA2, PTF1A, HNF6, and NKX6.1 were increased, whereas the expression of intestinal marker CDX2 and liver marker AFP were suppressed (26). In addition, they also found that ILV and PKC agonists, TPB or PMA, can synergize with FGF10 signaling to promote the proliferation of PDX1^+^ cells derived from hPSCs. Interestingly, ILV treatment also works on mouse embryonic stem cells, suggesting a conservation of signaling pathway in both mouse and human cells (26) (Table 1).

Subsequently, Rezania et al. (4) employed two PKC activators in the mid-late stage of hPSC differentiation; 50 nM TPB (safer profile) and phorbol 12,13-dibutyrate (PDBu) (27), demonstrating that the activation of PKC signaling induces the gene expression of pancreatic lineage markers *PTF1A*, *NGN3*, *NEUROD1*, and *NKX6.1* while suppressing the expression of intestinal (*CDX2*) and liver (*ALB*) markers (4). This demonstrated that PKC signaling enriches the development of pancreatic progenitors while inhibiting intestinal and hepatic lineages. Similarly, Pagliuca et al. used 500 nM PDBu in their pancreatic differentiation protocol (7) (Figure 1B; Table 1). Although these data are encouraging, more studies remain to be done to thoroughly clarify the role of PKC signaling and the specific mechanisms in the maturation of pancreatic endocrine progenitors.

## Lower Level of Retinoic Acid Signaling as Pancreatic Differentiation Progresses

It is well-established that RA signaling plays critical roles in the early and late stages of pancreas development (28). RA is a lipid-soluble vitamin A derivative synthesized from the oxidation of retinaldehyde via enzymes retinaldehyde dehydrogenase 1 (RALDH1), RALDH2, and RALDH3. RA produced at the splanchnic lateral plate mesoderm and Raldh2 expressed in the dorsal pancreatic mesenchyme promote Pdx1 induction in the dorsal foregut endoderm (Figure 1A). Raldh2 mutant mice exhibit dorsal pancreatic bud agenesis (29) as they fail to form pancreatic progenitors, indicated by the loss of Pdx1, Prox1, altered Isl1, and reduced Hlxb9 expression (29, 30).

Many existing protocols differentiating hPSCs into pancreatic cells utilize 1–3 μM RA. As an alternative, Schulz et al. replaced RA with 3 nM TTNPB/arotinoid acid – a more stable retinoid analog that can selectively activate RA receptors (RARs) (17). Interestingly, Rezania et al. started out using 1 μM RA during the early posterior foregut differentiation, subsequently reducing to 100 nM RA during pancreatic endoderm phase and further reducing to 50 nM during the pancreatic endocrine phase (6). Pagliuca et al. also reported a similar pancreatic differentiation protocol in which a decreasing dose of RA was used, starting with 2 μM followed by 100 nM RA at later stages, which was eventually reduced to 25 nM (7) (Figure 1B; Table 1).

While it is widely accepted that RA is crucial for pancreatic specification, using progressively lower doses of RA as practiced by Rezania et al. (6) and Pagliuca et al. (7) raises questions about the importance of RA concentrations during pancreas development both *in vitro* and *in vivo*. RA signals by binding RARs and retinoid X receptors (RXRs); so it is postulated that a decrease in RA signaling may be more conducive for subsequent pancreatic endocrine specification since retinoid receptors are upregulated in the pancreatic exocrine (31). However, given the lack of understanding in this regard, the significance of the dose of RA during human pancreas development warrants further studies.

## Inhibiting γ-Secretase/Notch for Accelerated Pancreatic Endocrine Differentiation

Notch signaling is essential for the proper development of pancreatic endocrine progenitors as it regulates their decision between differentiation and proliferation (32). The reduction of Notch signaling is known to promote accelerated pancreatic endocrine differentiation (33). Similarly, the inhibition of Notch signaling via γ-secretase (an intra-membrane protease) inhibitor can downregulate the expression of Notch target Hes-1, an inhibitor of pro-endocrine gene Ngn3 (34) (Figure 1A). Conversely, the activation of Notch in PDX1^+^ pancreatic progenitors prevents pancreatic differentiation (35).

*N*-[*N*-(3,5-difluorophenacetyl-l-alanyl)]-*S*-phenylglycine t-butyl ester (DAPT) is a commonly used γ-secretase inhibitor with an IC50 in the nM range. The inhibition of Notch signaling with DAPT can increase Ngn3 mRNA and protein expression in adult islets (36). Likewise, DAPT increases *NGN3* and *NEUROD1* gene expression in hPSC-derived pancreatic progenitors (18). In recent protocols developed by Pagliuca et al. (7) and Rezania et al. (6), other γ-secretase inhibitors have been employed to retard Notch signaling. Rezania et al. used γ-secretase inhibitor XX (GSiXX) that has an IC50 in the low nM range. They showed that GSiXX can induce the expression of β cell maturation genes but inhibit the expression of *PTF1A*, a marker of pancreatic exocrine lineage (6). GSiXX can also act in concert with triiodothyronine (T3) to increase the percentage of NKX6.1^+^INS^+^GCG^−^ β-like cells (6). Alternatively, Pagliuca et al. employed the use of XXI at 1 μM (7), which has an IC50 in the picomolar range, and demonstrated that XXI worked with other factors to improve β cell gene expression (Figure 1B; Table 1). However, it remains unclear whether there are differences between DAPT, GSIXX, or XXI in the induction or suppression of key pancreatic transcription factors for the eventual promotion of pancreatic β cell formation.

## Triiodothyronine Could Promote Pancreatic β Cell Maturation

Studies in the 1980s suggest that thyroid hormones regulate insulin secretion, possibly via control over glucose oxidation and calcium uptake rates (37). T3, a thyroid hormone, can potentiate insulin signaling and increase insulin synthesis in diabetic rodents (38), in rodent islets (39), and in a rodent pancreatic β cell line (40) (Figure 1A). Mechanistically, T3 phosphorylates and activates AKT in pancreatic β cells, improving their survival (39, 41); 0.1 μM of T3 can increase rodent pancreatic β cell proliferation via the MAPK/extracellular signal-regulated kinase (ERK) signaling pathway (42). Interestingly, T3 apparently induces the transdifferentiation of human pancreatic ductal cell line (hPANC-1) into β-like cells, with an increased expression of *INS* transcripts (43). T3 can also increase both the mRNA expression of pro-endocrine gene Ngn3 and the number of β cells, indirectly inducing endocrine differentiation from exocrine cells; possibly via Akt signaling (44).

Of late, T3 has been shown to promote pancreatic β cell maturation and proliferation in rats (45). Based on these findings, Rezania et al. went on to demonstrate that 1 μM of T3 can actually induce the expression of *INS* and mature β cell markers, and enhance the co-expression of NKX6.1 and INSULIN protein (6). Similarly, Pagliuca et al. employed the same dose of 1 μM T3 in the later stages of their pancreatic differentiation protocol to generate human pancreatic β cells from hPSCs (7) (Figure 1B; Table 1). While the biology and role of T3 in pancreatic β cell maturation remains to be explored further, its inclusion in pancreatic differentiation protocols appears to serve a positive function.

## Inhibition of Tyrosine Kinase Receptor Axl Induces Mature Pancreatic β Cell Marker (Mafa) Expression

AXL is a member of the Tyro3-Axl-Mer (TAM) trans-membrane receptor tyrosine kinase (RTK) family that plays an important role in essential cellular processes, such as cell survival, growth, proliferation, and differentiation. Its ligand, growth arrest specific 6 (Gas6), binds AXL to activate downstream signaling, including the phosphoinositide 3-kinase (PI3K), ERK, and signal transducer and activator of transcription 3 (STAT3) signaling (46). Interestingly, Rezania et al. performed small molecule and growth factor library screening to identify compounds that can induce mature β cell marker MAFA from hPSC-derived pancreatic progenitors and found that 2 μM BGB324 (R428), an inhibitor of AXL, can induce MAFA protein expression (6) (Figure 1B; Table 1). However, there is little information linking AXL to pancreas development and β cell maturation.

In 1999, Augustine et al. reported that the overexpression of AXL results in diabetes in mice. Furthermore, the administration of exogenous Gas6 exacerbated the condition (47). Haase et al. recently confirmed that GAS6 is expressed in pancreatic tissues and found that GAS6 reduced *Mafa* gene expression in rodents (48), likely due to the activation of AXL (Figure 1A). This corresponds with the increase in MAFA expression observed by Rezania et al. after the inhibition of AXL signaling (6). While there seems to be a *bona fide* association between AXL signaling and pancreas development, the cellular mechanism(s) remain a mystery.

## Antioxidants may Benefit Pancreatic Differentiation

Excessive levels of reactive oxygen species (ROS) have been implicated in glucotoxicity-induced pancreatic β cell destruction and dysfunction. In this regard, antioxidants play important defensive roles against ROS. In the endocrine pancreas, the antioxidant vitamin C is known to be an effective co-factor for the peptidyl α-amidation of several biologically active peptides and is necessary for optimal insulin secretion from pancreatic β cells (Figure 1A). In fact, high concentrations of ascorbic acid (vitamin C) were found in neonatal rat endocrine pancreas (49). Mechanistic studies involving other antioxidants, such as glutathione peroxidase-1 (GPx-1), *N*-acetylcysteine (NAC), and ebselen, were reported to maintain the protein expression of mature β cell marker MAFA (20–22) (Table 1).

Interestingly, Rezania et al. used 1–2 mM NAC during their pancreatic differentiation and found that it also increased nuclear MAFA protein expression (6). However, this was not replicated with another antioxidant, vitamin E. Pagliuca et al. also relied upon the use of 0.25 mM of vitamin C throughout S1–S5 phase of their pancreatic differentiation protocol to generate mature and functional human pancreatic β cells (7) (Figure 1B; Table 1). While the metabolism of vitamins C and E are altered before the onset of diabetes in rats, their contribution to the pancreas is unclear (50). Antioxidant treatments may preserve β cell function, exerting positive effects in diabetes (51), but their role in pancreas development and β cell maturation certainly remains elusive.

## Betacellulin Directs a Pancreatic β Cell Fate

Betacellulin (BTC) is a member of the epidermal growth factor (EGF) family that plays a role in the differentiation of pancreatic β cells (52) (Figure 1A). It is largely expressed in the liver, kidney, small intestine, and pancreas (53), and is specifically expressed in 9- to 24-week-old human fetal pancreas (54). BTC binds to ErbB-1 and ErbB-4 receptors (55) to initiate downstream signaling pathways involving PI3K/Akt, MAPK, STAT, and mTOR signaling pathways (56).

Betacellulin appears to direct a pancreatic β cell fate. It can convert exocrine cells (57) and α cells (58) into insulin-secreting cells. It can also induce β cell neogenesis from ductal cells in diabetic mice (59). Li et al. demonstrated that exogenous BTC can promote β cell regeneration in 90% pancreatectomized rats (60) and convert δ to β cells in STZ-induced diabetic mice (61). Also, Yamamoto et al. observed that long-term administration of BTC reverses STZ-induced hyperglycemia in mice (62). Surprisingly, the loss of BTC in mice yielded no overt defect (63) despite their active roles in the pancreas. This could be explained by compensatory effects exhibited by the other EGFR ligands (64). The overexpression of BTC in transgenic islets does not affect islet structure, endocrine cell ratio, or β cell mass but enhances glucose-stimulated insulin secretion (65). However, ubiquitous overexpression of BTC in mice results in various pathologies (66). Intriguingly, gene variants and polymorphisms in the *BTC* gene have also been found to be associated with types 1 and 2 diabetes (54, 67).

The induction of β cell development/differentiation by BTC (52) could be an outcome of the downstream increase in insulin receptor substrate-2 (IRS-2) expression (68), an important mediator of β cell function (69). BTC can induce *Pax4* gene expression in rat islets, promoting β cell functionality (70). It can also sustain *PDX1* expression and induce β cell differentiation from hPSCs (71). Ten ng/ml BTC are sufficient to increase *Pdx1* gene expression, insulin production, and to inhibit amylase and glucagon production in mouse embryonic pancreas explants (72). Lately, Pagliuca et al. employed 20 ng/ml BTC in the later stages of their pancreatic differentiation protocol (7). Similarly, 50 ng/ml EGF was added to preserve cell mass (17) (Figure 1B; Table 1). These data strongly indicate that BTC and/or other EGF ligands are of importance in pancreas development and β cell maturation. Nonetheless, more detailed molecular mechanisms remain to be uncovered.

## Concluding Remarks

Relating to the current most advanced human pancreatic β cell differentiation protocols, the biological mechanisms involved in the later stages of β cell development and maturation still remain elusive. The fact that the postnatal stage in rodents is grossly equivalent to the third trimester in human pancreas development (73) invites new approaches to study this aspect of β cell biology. In this review, we revisited some of the least-understood developmental signaling pathways in rodent pancreas biology. Our efforts unraveled interesting aspects of these signaling pathways that demand to be thoroughly elucidated at the mechanistic level. Future studies should seek to highlight how immature β cells transit into mature and functional β cells. This would certainly advance our knowledge of human pancreas developmental biology and boost translational efforts in the use of stem cells for diabetes treatment.

## Author Contributions

MS: reviewed the literature, wrote and edited the paper. BL: edited the paper. NP: edited the paper and prepared the figure. AT: conceptualized the review topic and contents, wrote, edited, and approved the paper.

## Conflict of Interest Statement

The authors declare that the research was conducted in the absence of any commercial or financial relationships that could be construed as a potential conflict of interest.
